# Negligible Immunogenicity of Induced Pluripotent Stem Cells Derived from Human Skin Fibroblasts

**DOI:** 10.1371/journal.pone.0114949

**Published:** 2014-12-11

**Authors:** Qiao Lu, Meixing Yu, Chongyang Shen, Xiaoping Chen, Ting Feng, Yongchao Yao, Jinrong Li, Hong Li, Wenwei Tu

**Affiliations:** 1 The Joint Research Center of West China Second University Hospital of Sichuan University and Faculty of Medicine of the University of Hong Kong, Sichuan University, Chengdu, Sichuan, 610041, China; 2 Department of Pediatrics, University Hospital of Hubei University for Nationalities, Enshi, Hubei, 445000, China; 3 Laboratory of Pathogen Biology, State Key Laboratory of Respiratory Disease, Center for Infection and Immunity, Guangzhou Institutes of Biomedicine and Health, Chinese Academy of Sciences, Guangzhou, 510530, China; 4 Key Laboratory of Obstetric and Gynecologic and Pediatric Diseases and Birth Defects of Ministry of Education, West China Second University Hospital, Sichuan University, Chengdu, Sichuan, 610041, China; 5 Department of Pediatrics and Adolescent Medicine, Li Ka Shing Faculty of Medicine, The University of Hong Kong, Pokfulam, Hong Kong SAR, China; Department of Immunology, China

## Abstract

Human induced pluripotent stem cells (hiPSCs) have potential applications in cell replacement therapy and regenerative medicine. However, limited information is available regarding the immunologic features of iPSCs. In this study, expression of MHC and T cell co-stimulatory molecules in hiPSCs, and the effects on activation, proliferation and cytokine production in allogeneic human peripheral blood mononuclear cells were examined. We found that no-integrate hiPSCs had no MHC-II and T cell co-stimulatory molecules expressions but had moderate level of MHC-I and HLA-G expressions. In contrast to human skin fibroblasts (HSFs) which significantly induced allogeneic T cell activation and proliferation, hiPSCs failed to induce allogeneic CD45^+^ lymphocyte and CD8^+^ T cell activation and proliferation but could induce a low level of allogeneic CD4^+^ T cell proliferation. Unlike HSFs which induced allogeneic lymphocytes to produce high levels of IFN-γ, TNF-α and IL-17, hiPSCs only induced allogeneic lymphocytes to produce IL-2 and IL-10, and promote IL-10-secreting regulatory T cell (Treg) generation. Our study suggests that the integration-free hiPSCs had low or negligible immunogenicity, which may result from their induction of IL-10-secreting Treg.

## Introduction

Development of innovative strategies to prevent allograft rejection is a focus of transplantation medicine. In addition to solid organ transplantation, cellular transplantation involved in tissue restoration should take into account the potential for rejection and need to induce immune tolerance [1]. The successful isolation of human embryonic stem cells (hESCs) provided a valuable source for cell replacement therapy [2]. Various studies have confirmed that hESCs have powerful therapeutic potential [3]–[6]. However, hESC-based therapy is associated with ethical challenges. The recent groundbreaking invention of induced pluripotent stem cells (iPSCs) contribute to an alternative candidate for regenerative medicine.

iPSCs reprogrammed from somatic cells with defined factors have similar features to ESCs, which can self-renew and be differentiated into various cell types of all 3 germ layers *in vitro* and *in vivo* [7], [8]. Cells differentiated from iPSCs also have the capacity to replace the biological functions of various organs, as shown in animal models of Parkinson's disease [9], sickle-cell anaemia [10], spinal cord injury [11], and myocardial infarction [12]. In contrast to ESCs, patient-specific iPSCs can be generated without ethical issues. With the development of reprogramming techniques, various somatic cell types from different species and tissues have been successfully induced to iPSCs [12]–[16]. Therefore, the potential advantage of iPSCs in biomedical research is revealed. However, the efficiency, stability, safety, and immunogenicity of iPSCs should be assessed prior to clinical application.

It is widely assumed that autologous iPSCs and their derivatives should be immunologically tolerated by the recipient. However, this dogma was challenged by a study showing T-cell-dependent immune rejection of syngeneic mouse iPSCs (miPSCs) following transplantation [17], in which miPSCs derived via the episomal approach were less prone to immune-mediated attack than those generated using viral vectors. Another study showed that short-term immunosuppression by inhibiting leukocyte co-stimulatory molecules promoted engraftment of embryonic and induced pluripotent stem cells [18]. Recently, it was reported that low immunogenicity of less immunogenic cells could be retained after cell reprogramming and further differentiation [19]. Nevertheless, another finding has demonstrated limited or no immune response including T cell infiltration in tissues derived from autologous iPSCs or allogenic ES cells [20]. Interestingly, it was shown that hiPSCs-derived CD34^+^ hematopoietic progenitor cells (HPCs) expressed HLA-G and could induce T cell anergy [21]. Therefore, the immunogenicity of hiPSCs and their derivatives remains unclear and needs to be further examined.

In addition to application of terminally differentiated cells derived from iPSCs in CRT, undifferentiated iPSCs could be used for vaccination after modification with target genes [22]. From a practical point of view, generation of the required cell type for each patient is time- and cost-intensive [23]. Thus, the use of allogeneic iPS cell lines is expected be preferable, and it is necessary to research the immunological characteristics of allogeneic hiPSCs. In this study, we examined MHC and T cell co-stimulatory molecules in hiPSCs derived from skin fibroblasts, and further determined their effects on activation, proliferation and cytokine production in allogeneic human peripheral blood mononuclear cells.

## Results

### Characterization of hiPSCs

**Figure 1:**
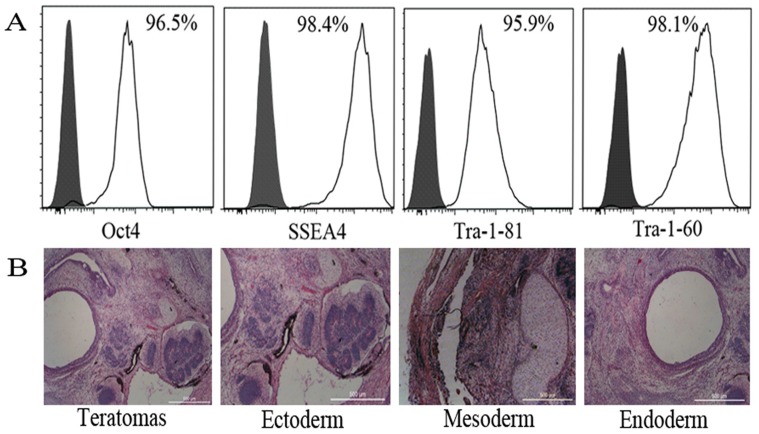
Characterization of hiPSCs. (A) Expression of human ES cell-specific cell surface markers on hiPSCs were analyzed by flow cytometry. Gray histograms: isotype controls; White histograms: positive staining of antigens. (B) Various tissues of all three germ layers present in teratomas derived from hiPSCs. Hematoxylin and eosin staining of teratoma sections. Scale bars, 500 µm.

To demonstrate the quality and undifferentiated phenotype of hiPSCs used in this study, the expressions of OCT4, SSEA-4, TRA-1-60, and TRA-1-81 in hiPSCs were analyzed by flow cytometry; more than 95% of hiPSCs were positive for these markers (Fig. 1A). The pluripotency of this cell line was also confirmed by its ability to form teratomas *in vivo* (Fig. 1B).

### Expression of MHC proteins and costimulatory molecules in hiPSCs

**Figure 2:**
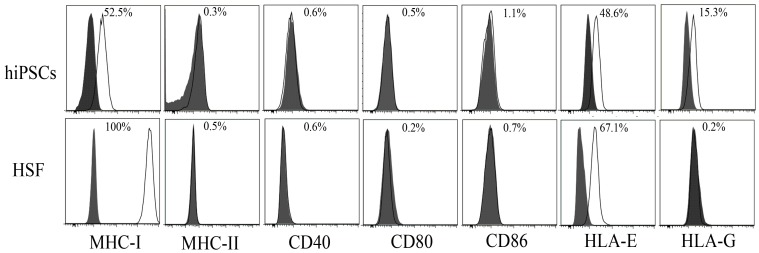
Phenotypes of hiPSCs and somatic cells (HSFs). White histograms represent the surface expression of MHC-I, MHC-II, HLA-G, HLA-E, CD40, CD80, and CD86, and gray histograms represent isotype controls. Data shown here are representative of three different experiments.

Nearly all nucleated cells express MHC-I antigens, whereas expression of MHC-II molecules is more restricted. MHC expression has been shown to be suppressed after cell reprogramming [24]. As shown in Fig. 2, significantly lower expression of MHC-I proteins was observed in hiPSCs compared with HSFs, but no MHC-II expression was observed in both cells. In addition to classical MHC proteins, we further analyzed the expression of non-classical MHC-I antigens (HLA-E and HLA-G) in hiPSCs. hiPSCs expressed moderate level of HLA-E, although the level is lower than that in HSFs. hiPSCs expressed low level of HLA-G, whereas there was no HLA-G expression in HSFs (Fig. 2). In addition, we also examined the costimulatory molecules in hiPSC and HSFs, and found there were no CD80, CD86, and CD40 expression in these cells (Fig. 2).

### Effects of IFN-γ on MHC protein and co-stimulatory molecule expression in hiPSCs

**Figure 3:**
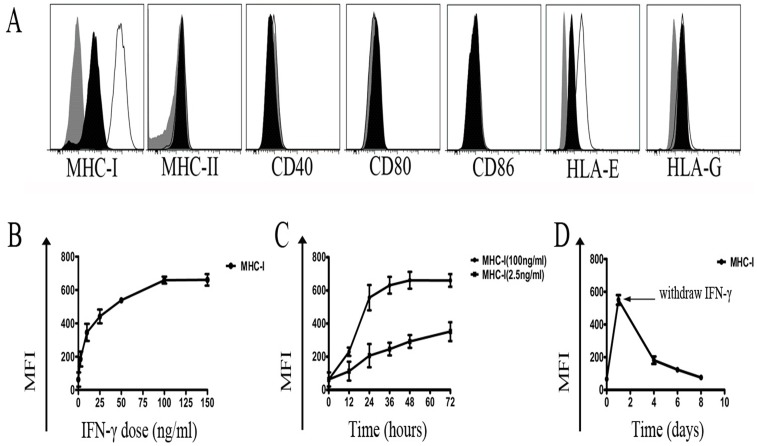
Effect of IFN-γ on hiPSCs. (A) Expression of MHC proteins and costimulatory molecules by hiPSCs treated with 100 ng/ml of IFN-γ for 48h. Gray: isotype controls; Black: positive staining of antigens expressed on hiPSCs without IFN-γ treatment; White: positive staining of antigens expressed on hiPSCs treated with IFN-γ. (B) Concentration dependence of MHC-I induction by IFN-γ in hiPSCs. (C) Time response of MHC-I expression in hiPSCs treated with IFN-γ. (D) MHC-I expression gradually decreased after IFN-γ was withdrawn from the culture medium. Three independent experiments were performed for each analysis.

IFN-γ is known to increase the expression of MHC-I and MHC-II proteins. It has been reported that IFN-γ can induce the expression of HLA-A/B/C and β2M in human ES cells [25]. To determine whether IFN-γ influences the expression of MHC and costimulatory molecules in hiPSCs, we analyzed MHC expression upon IFN-γ treatment. As shown in Fig. 3A, no significant change in the expression of MHC-II, CD40, CD80, CD86, and HLA-G was observed in the hiPSCs after addition of 2.5 ng/ml to 150 ng/ml IFN-γ to the growth medium for 48 hours. In contrast, IFN-γ significantly upregulated MHC-I and HLA-E expressions in hiPSCs. The upregulation of MHC-I expression by IFN-γ was showed in the dose- and time-dependent manner (Fig. 3B and C). Even 2.5 ng/ml of IFN-γ could result in a dramatic elevation in MHC-I expression and the maximal expression was observed after treatment with 100 ng/ml of IFN-γ for 48 hours. A remarkable decline in MHC-I expression was observed when IFN-γ was withdrawn from the growth medium (Fig. 3D).

### hiPSCs do not effectively induce activation and proliferation of allogeneic lymphocytes

**Figure 4:**
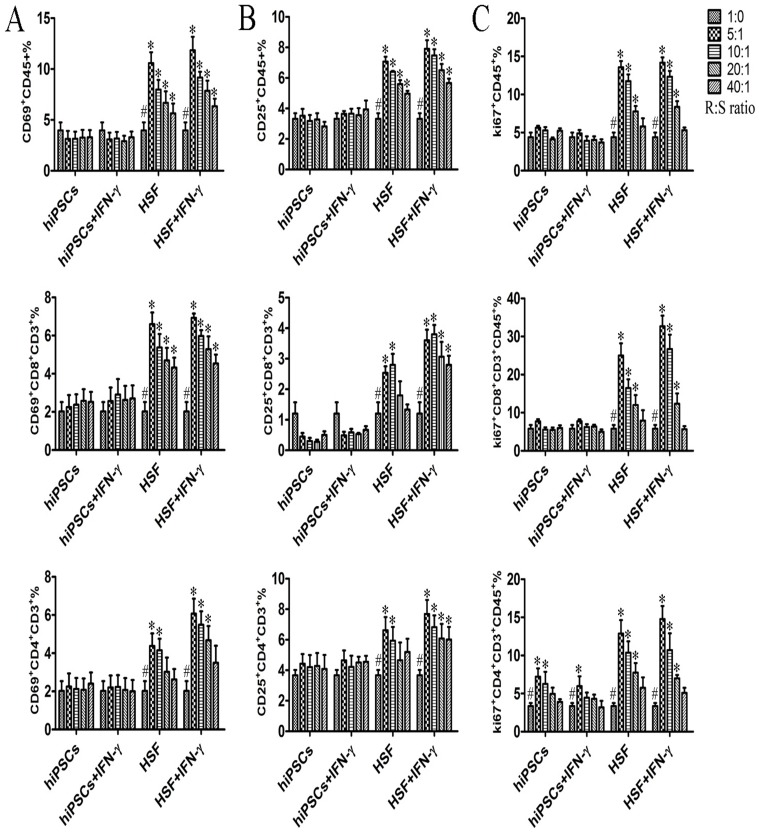
hiPSCs do not effectively induce activation and proliferation responses on allogeneic lymphocytes. hiPSCs pretreated with or without IFN-γ (stimulator cells; S) were inactivated and then directly cultured with allogeneic PBMCs (responder cells; R) at different R/S cell ratios in a MLR for 5–7 days (n = 4). PBMCs were harvested for examination of the expression of activation markers and Ki67 protein at the indicated time point. Surface expression of CD69 and CD25 on PBMCs, CD3^+^CD8^+^ T cells, and CD3^+^CD4^+^ T cells was measured by flow cytometry after 6 h and 24 h of co-culture (A, B). (C) Intranuclear Ki67 protein expression in PBMCs, CD3^+^CD8^+^ T cells and CD3^+^CD4^+^ T cells was analyzed after 5–7 d of stimulation. Data are shown as the mean ± SEM. Results are representative of four different experiments. #, indicating significant difference compared to those with different ratio of stimulator within same group (p<0.05). *, indicating significant difference compared to those with same and same ratio of stimulator (p<0.05).

To determine whether hiPSCs could induce a proliferative response on allogeneic lymphocytes, we first assessed the effect of hiPSCs on PBMCs activation. Fresh PBMCs were isolated and then directly co-cultured with different numbers of hiPSCs. Subsequently, PBMCs were examined for the expression of surface activation markers CD69 and CD25. As shown in Fig. 4A and 4B, HSFs significantly increase the CD69 and CD25 expressions in allogeneic CD45^+^ lymphocytes and CD4^+^ and CD8^+^ T cells in a dose-dependent manner as expected, whereas hiPSCs did not increase the surface expression of CD69 and CD25 in CD45^+^ lymphocytes and CD4^+^ and CD8^+^ T cells.

We then evaluated the lymphocyte proliferation induced by allogeneic hiPSCs and HSFs by determining the Ki67 expression. PBMCs used as responder cells (R) were cultured with mitomycin-treated hiPSCs (stimulator, S) at different R:S ratio. As shown in Fig. 4c, hiPSCs failed to induce allogeneic CD45^+^ lymphocytes and CD8^+^ T cell proliferation, whereas mitomycin-treated HSFs significantly induced allogeneic CD45^+^ lymphocytes and CD8 T cell proliferation. Interestingly, we found that hiPSCs could induce CD4^+^ T cell proliferation although the increase of CD4^+^ T cell proliferation induced by hiPSCs was much lower than that induced by HSFs (Fig. 4C). To determine whether the non-responsiveness of allgeneic lymphocytes to hiPSCs was due to the cell death during MLR, we further examined the viability of PBMCs after MLR reaction by Trypan blue staining and found that no significant death of PBMCs caused by hiPSCs (data not shown).

### IFN-γ does not induce the activation and proliferation of allogeneic lymphocytes to hiPSCs

As IFN-γ could increase MHC-I and HLA-E expressions in hiPSCs (Fig. 3A), we further examined the effect of IFN-γ-pre-treated hiPSCs on the activation and proliferation of allogeneic PBMCs during MLR. As shown in Fig. 4, there were no significant differences of the activation and proliferation in allogeneic CD45+ lymphocytes, CD4^+^ and CD8^+^ T cells upon hiPSCs versus IFN-γ-pre-treated hiPSCs stimulations in terms of CD69, CD25 and Ki67 expressions. Similar results were also found when allogeneic PBMCs were stimulated with HSFs versus IFN-γ-pre-treated HSFs (Fig. 4).

### hiPSCs induce allogeneic PBMCs to produce IL-10 and IL-2

**Figure 5:**
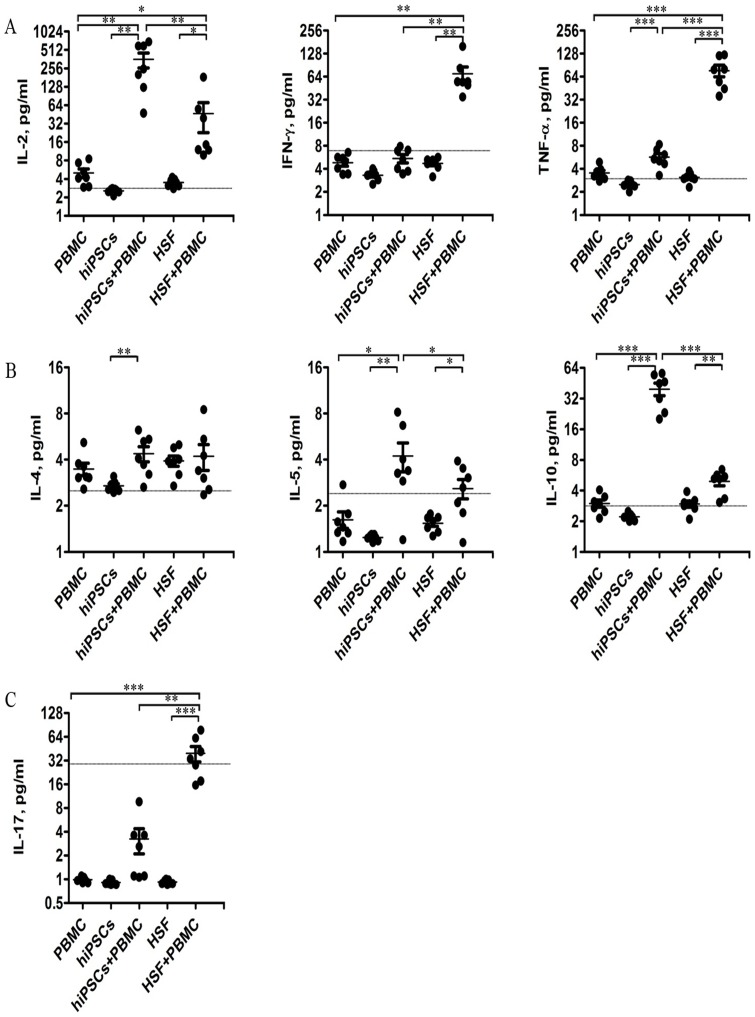
Cytokine expression profile in MLR. Culture supernatants were collected from the MLR after 24 hours of culture and examined for expression of cytokines secreted by Th1 (A), Th2 (B), and Th17 (C) (n = 7). Data shown are single values for each point in the scatter plot. * *P<*0.05; ** P<0.01; *** P<0.001. The grey lines indicate the sensitivity of each cytokine.

**Figure 6:**
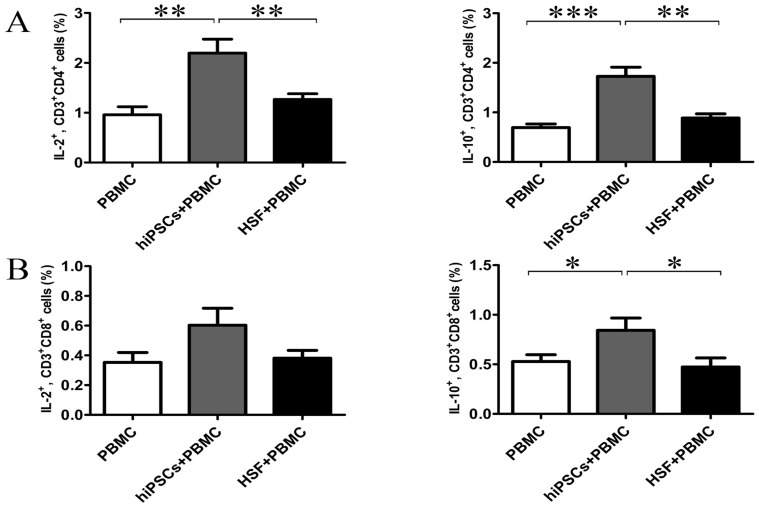
hiPSCs induced the generation of IL-2- and IL-10-secreting T cells in MRL. Allogeneic PBMCs (responder cells; R) were co-cultured with hiPSCs (stimulator cells; S) at an R/S ratio of 10∶1 for 24 hours. The intracellular expressions of IL-2 and IL-10 in CD3^+^CD4^+^ and CD3^+^CD8^+^ T cells were examined by flow cytometry (A, B) (n = 7). Data shown as the mean ± SEM are representative of seven separate experiments. * *P<*0.05; ** P<0.01; *** P<0.001.

To determine the mechanism underlying the non-responsiveness of allgeneic lymphocytes to hiPSCs, we further determined IL-2, IFN-γ, TNF-α, IL-4, IL-5, IL-10 and IL-17 productions in the supernatants after 24 hours of co-culture of allogeneic PBMCs with hiPSCs or HSFs. As shown in Fig. 5, PBMCs, hiPSCs or HSFs alone had little or no these cytokine productions. HSFs could induce PBMCs to produce a large amount of IFN-γ and TNF-α, but only a little of IL-2, IL-5, IL-10 and IL-17. In contrast, hiPSCs could induce PBMCs to produce a large amount of IL-2 and IL-10, but a little or no IFN-γ, TNF-α, IL-4, IL-5 and IL-17 production. To further confirm these results, we further determined the cytokine secreting T cells at single cell level. Consistent with the cytokine production, hiPSCs increased the percentage of IL-2- and IL-10-secreting CD4 and CD8 T cells in PBMCs, although the increase of the percentage of IL-2-secreting CD8 T cells in PBMCs did not reach the statistical difference (Fig. 6). In contrast, HSFs did not increase the percentage of IL-2- and IL-10-secreting CD4 and CD8 T cells (Fig. 6).

## Discussion

iPSCs have a great promise for cell-based transplantation and regenerative medicine, however, there is only very limited information available regarding the immunologic features of iPSCs. The different pluripotent stem cell lines may have different immunogenicity because of the difference in the methods of reprogramming, cell sources, and culture conditions. In the current study, hiPSCs prepared without transgene integration were maintained in a feeder-free culture system. We analyzed the immunogenic characteristics of these cells in comparison to somatic cells (HSFs) and investigated their immune response *in vitro*. Our data demonstrated that hiPSCs have not obviously immunogenicity in terms of inducing allogeneic lymphocyte activation and proliferation. More importantly, unlike the somatic cell line (HSFs) which mainly induce allogeneic PBMCs to produce Th1 type cytokines, such as IFN-γ and TNF-α, here we demonstrated that hiPSCs can induce allogeneic T cells to produce regulatory cytokine IL-10. This, to the best of our knowledge, is the first report to determine that hiPSCs can induce IL-10 producing regulatory T cells.

It is known that expression of MHC antigens in tissues determines the outcome of alloantigen-specific T cell responses *in vitro* and *in vivo* [26]. The low immunogenicity of ESCs results from low expression of MHC-I and negative MHC-II, costimulatory molecules [25], [27], [28]. Consistent with a previous report that human iPSCs have low MHC-I gene expression, un-detectable MHC-II expression [29], here we confirmed that non-integration hiPSCs also had relatively low classical MHC-I expression, and no expressions of MHC-II and co-stimulatory molecules, suggesting the hiPSCs have a limited capacity for antigen processing and presentation. As the promoter of MHC-I contains the interferon-stimulated response element, IFN-γ can induce the expression of HLA-A/B/C and β2M in human ES cells [25]. Consistent with this, here we also found that IFN-γ could significantly enhance the MHC-I expression in hiPSCs, suggesting IFN-γ may induce the activation and proliferation of allogeneic lymphocytes to hiPSCs. However, we did not found any effect of IFN-γ on the activation and proliferation of allogeneic lymphocytes to hiPSCs. The reason for this may be related to the expression of HLA-G on the hiPSCs and the induction of IL-10 after co-culture of hiPSCs with allogeneic T cells.

HLA-G is present on extra-embryonic trophectoderm cells and associated with protection of the allogeneic fetus from the maternal immune system [30]. HLA-G is believed to contribute to the immune regulatory function of hESCs and MSC because HLA-G is also expressed in these stem cells [31], [32]. Interestingly, here we found for the first time that hiPSCs expressed a moderate level of HLA-G, indicating our hiPSCs may have immune regulatory capacity. Indeed, we found that hiPSCs can induce allogeneic T cells to produce regulatory cytokine IL-10.

Host immune system activation and clonal proliferation of effector lymphocytes are critical for graft rejection. We examined the effects of PBMCs on hiPSCs in MLR. Our data demonstrated that hiPSCs did not induce activation of allogeneic lymphocytes, including their subpopulations; furthermore, this response was not amplified with an increased quantity of allo-antigens. Importantly, we found that hiPSCs did not stimulate the proliferative response in total CD45^+^ lymphocytes. Additionally, no proliferation was detected in CD8^+^ T cells even at higher levels of stimulator cells, probably because of their low MHC-I expression and lack of costimulatory molecules in hiPSCs. Interestingly, hiPSCs induced CD4^+^ T cell proliferation although the proliferation of CD4^+^ T cells was much lower than that induced by HSFs. Indeed, here we further found that hiPSCs can induce allogeneic T cells to produce regulatory cytokine IL-10, and induce the generation of IL-10-secreting Treg, which may be account for the low or negligible immunogenicity of the hiPSCs.

The role of cytokines in allograft rejection and induction of transplantation tolerance has been largely studied in the context of the Th1/Th2/Treg/Th17 paradigm. IFN-γ and TNF-α are mainly produced by Th1 cells and contribute to the graft rejection [33]. IL-2 is critical for the development and peripheral expansion of Treg, which promote self-tolerance by suppressing T cell responses [34]–[36]. IL-10 secreted from Treg cells is an immunosuppressive cytokine which can prevent graft rejection [37]. IL-17a plays an important role in chronic inflammation, auto-immune diseases, and immune rejection [38]–[40]. Here we demonstrated that hiPSCs can induce allogeneic PBMCs to produce high level of IL-2 and moderate level of IL-10, but are failed to produce IFN-γ, TNF-α and IL-17, thus leading to the low or negligible immunogenicity of the hiPSCs.

In conclusion, we demonstrate that hiPSCs prepared without transgene integration have low or negligible immunogenicity. They have low expression of MHC-I, but no expression of MHC-II or the costimulatory molecules such as CD80, CD86, and CD40. We also found that hiPSCs do not initiate allogenic CD45^+^ lymphocyte and CD8^+^ T cell proliferation and activation *in vitro*. Moreover, our data revealed for the first time that these hiPSCs may have immunomodulatory properties because they can promote the generation of IL-10-secreting Treg, which may be valuable for their potential clinical applications.

## Materials and Methods

This study was conducted in compliance with the Declaration of Helsinki, and the research protocol was approved by the Institutional Review Board of the West China Second University Hospital of Sichuan University. All participants provided their written informed consent to participate in this study.

### Cell culture

This hiPSC line was derived from human skin fibroblasts donated by a healthy volunteer without integration of the vector and transgene sequences and fully characterized [41], [42], and maintained in a feeder-free culture system using mTesR1 (Stem Cell Technologies) and Matrigel (BD Bioscience). Human skin fibroblasts (HSFs) were established as the parental somatic cell control and cultured as previously described [43].

### Human induced pluripotent stem cells identification

Undifferentiated hiPSCs were used at passages 30–45. Molecules specifically expressed by stem cells including membrane antigens (SSEA4, TRA-1-60, and TRA-1-81) and octamer-binding nuclear transcriptional factor 4 (OCT4) were identified by flow cytometry. For teratoma formation, 2×10^6^ cells were injected into the subcutaneous space in both dorsolateral areas of 4-week-old nude mice (5 mice were used). After 6-8 weeks, teratomas were removed, fixed, and embedded. Paraffin-embedded teratomas were cut into serial sections, stained with hematoxylin-eosin, and observed under a microscope.

### Flow cytometry

For surface marker expression, cells were stained with the following antibodies as we did before [44]: mouse anti-SSEA4, anti-TRA-1-60, and anti-TRA-1-81 to identify hiPSCs; anti-CD45, anti-CD3, anti-CD4, anti-CD8, anti-CD69, and anti-CD25 to identify lymphocytes; and anti-MHC-I, anti-MHC-II, anti-HLA-E, anti-HLA-G, anti-CD80, anti-CD86 and anti-CD40 to determine hiPSCs immunophenotype. For intracellular staining, Intracellular staining was performed after cell fixation and permeabilization as we described before [45], and the following antibodies were used: anti-IL-2, IL-10, anti-OCT4 and anti-ki67 (Biolegend) or the relevant isotype controls. All samples were evaluated by flow cytometry (Beckman Coulter Gallios) and analyzed using FlowJo software (version 7.6.5, Tree Star, Inc.).

### IFN-γ treatment

Recombinant human IFN-γ (PeproTech) was reconstituted at a concentration of 25 µg/ml, aliquoted, and stored at −20°C. Cytokine treatments were performed as previously described [25]. Briefly, to determine the concentration response of hiPSCs to IFN-γ, the cytokine was added to the growth media at 2.5–150 ng/ml for 48 hours. For time course analysis, MHC-I expression was monitored 12–72 hours after addition of 100 ng/ml IFN-γ. In cytokine withdrawal experiments, the primary culture medium containing IFN-γ was discarded after 24 hours of culture, and cells were washed for three times with phosphate-buffered saline (PBS). Then, new medium lacking IFN-γ was added and protein expression was monitored 7 days after removal of IFN-γ.

### One-way mixed lymphocyte reaction (MLR)

Peripheral blood mononuclear cells (PBMCs) were isolated from buffy coats of blood contributed by normal healthy volunteers from Chengdu Blood Center. MLR was set as we described before [36], [46], [47]. Briefly, following Ficoll-Hypaque (Pharmacia) density gradient centrifugation, PBMCs were washed with PBS and re-suspended in serum-free medium, and used as responder. Human iPSCs or HSFs treated with or without IFN-γ were used as allogeneic stimulators. Then, responder cells (1×10^6^ cells/ml) were co-cultured with allogeneic stimulator cells at different ratios for 24 hours in 24-well flat-bottom plates, and responder cells alone were used as the control. Cells were collected and examined for CD69 expression after 6 hours and CD25 expression after 24 hours by flow cytometry respectively.

In PBMC proliferation experiments, the stimulator cells described above were pre-treated with mitomycin C (hiPSCs 3-5 µg/ml for 15 minutes at 37°C, HSFs 30 µg/ml for 2 hours at 37°C) prior to MLR. After 5–7 days of co-culture, non-adherent cells were harvested and stained with anti-CD45, anti-CD3, and anti-CD8 to identify lymphocytes and their subpopulations, and Ki67 to identify proliferating cells. Proliferation of allogeneic PBMCs was assessed by flow cytometry as the percentage of Ki67^+^ cells in CD45^+^ populations and their subsets as we did before [48]. The supernatant was also collected at the indicated time point and stored at −80°C for the subsequent experiments.

### Cytokine detection

Culture supernatants were collected in one-way mixed lymphocyte reactions. Cytokine (IL-2, IL-4, IL-5, IL-10, TNF-α, and IFN-γ) concentrations were measured in supernatants using a cytometric bead array (CBA; Becton & Dickinson) as we did before [35]. Data were acquired by flow cytometry and analyzed using CBA software. IL-17a was assayed by ELISA (R&D). Standard curves for each cytokine were generated to calculate the concentration in the tested samples.

### Statistical analysis

Data are presented as the mean ± standard error of the mean. Statistical significance was determined by one-way ANOVA or Student's *t*-test using SPSS 18.0 software. A *P* value of *<*0.05 was considered to be significant.
