# Correction: Negligible Immunogenicity of Induced Pluripotent Stem Cells Derived from Human Skin Fibroblasts

**DOI:** 10.1371/journal.pone.0120834

**Published:** 2015-03-05

**Authors:** The PLOS ONE Staff

The following information is missing from the Funding statement: Area of Excellence program (AoE/M-12/06), University Grants Committee of Hong Kong Special Administrative Region.

The complete, correct Funding statement is as follows: This work was supported in part by the grants from National Natural Science Foundation of China (NSFC) and the Research Grants Council (RGC) of Hong Kong Joint Research Scheme (N_HKU 747/11), National Natural Science Foundation of China (No. 81170606); Area of Excellence program (AoE/M-12/06), University Grants Committee of Hong Kong Special Administrative Region, and Doctoral Fund of Ministry of Education of China (No. 20120181110087). The funders had no role in study design, data collection and analysis, decision to publish, or preparation of the manuscript.
